# Potential Impacts of Climate Change on Native Plant Distributions in the Falkland Islands

**DOI:** 10.1371/journal.pone.0167026

**Published:** 2016-11-23

**Authors:** Rebecca Upson, Jennifer J. Williams, Tim P. Wilkinson, Colin P. Clubbe, Ilya M. D. Maclean, Jim H. McAdam, Justin F. Moat

**Affiliations:** 1 Royal Botanic Gardens, Kew, Richmond, United Kingdom; 2 Environment and Sustainability Institute, College of Life and Environmental Sciences, University of Exeter, Penryn Campus, Penryn, United Kingdom; 3 UK Falkland Islands Trust, Westminster, London, United Kingdom; 4 Agri Food and Biosciences Institute and Queens University of Belfast, New forge Lane, Belfast, Northern Ireland; 5 School of Geography, University of Nottingham, Nottingham, United Kingdom; Ecole Pratique des Hautes Etudes, FRANCE

## Abstract

The Falkland Islands are predicted to experience up to 2.2°C rise in mean annual temperature over the coming century, greater than four times the rate over the last century. Our study investigates likely vulnerabilities of a suite of range-restricted species whose distributions are associated with archipelago-wide climatic variation. We used present day climate maps calibrated using local weather data, 2020–2080 climate predictions from regional climate models, non-climate variables derived from a digital terrain model and a comprehensive database on local plant distributions. Weighted mean ensemble models were produced to assess changes in range sizes and overlaps between the current range and protected areas network. Target species included three globally threatened Falkland endemics, *Nassauvia falklandica*, *Nastanthus falklandicus* and *Plantago moorei*; and two nationally threatened species, *Acaena antarctica* and *Blechnum cordatum*. Our research demonstrates that temperature increases predicted for the next century have the potential to significantly alter plant distributions across the Falklands. Upland species, in particular, were found to be highly vulnerable to climate change impacts. No known locations of target upland species or the southwestern species *Plantago moorei* are predicted to remain environmentally suitable in the face of predicted climate change. We identify potential refugia for these species and associated gaps in the current protected areas network. Species currently restricted to the milder western parts of the archipelago are broadly predicted to expand their ranges under warmer temperatures. Our results emphasise the importance of implementing suitable adaptation strategies to offset climate change impacts, particularly site management. There is an urgent need for long-term monitoring and artificial warming experiments; the results of this study will inform the selection of the most suitable locations for these. Results are also helping inform management recommendations for the Falkland Islands Government who seek to better conserve their biodiversity and meet commitments to multi-lateral environmental agreements.

## Introduction

Island floras are particularly vulnerable to the impacts of climate change [1, 2, 3, 4, 5] and therefore understanding the likely responses is an urgent, if challenging, scientific problem. Situated in the South Atlantic, some 500 km from mainland South America, the Falkland Islands are a remote archipelago formed of two larger islands (East and West Falkland) and *c*. 500 smaller islands. In total, these islands cover an area of approximately 12,200 km^2^. The islands experience a cool temperate oceanic climate with mean temperatures for January and July of 9.4°C and 2.2°C, respectively [6]. Rainfall is low with a mean annual precipitation of 585 mm recorded at Mount Pleasant Complex on East Falkland during the periods 1987–2009 (data provided by UK Met Office).

The Falkland Islands has a relatively small native flora of 180 plant taxa (excluding one hybrid) including 14 endemic species [7]. The size of the flora suggests there is potentially little room for redundancy, increasing the importance of understanding which taxa are likely to be most at risk from the threats posed by climate change. Linking our understanding of the potential impacts of climate change on these vulnerable islands floras to policy recommendations is a critical element to ensure long-term conservation. The Aichi Biodiversity Targets recognise this at a global scale and provide a framework for policy action [8]. The Falkland Islands Government have developed a keen interest in how climate change might impact the Islands’ species, habitats and the ecosystems services they provide and are updating their Biodiversity Strategy accordingly [9].

Due to its position between the Antarctic and South American continents, the Falkland Islands contain plant species at the eastern and southern limits of their distribution, therefore likely to include climate change indicator species and unique genetic variation which is important to conserve in the face of changing climates and other threats [10, 11, 12]. Six endemic species are globally threatened, one near threatened and nationally, 22% (39 species) of the native vascular flora is at risk of extinction [7]. An understanding of how the risks associated with climate change will impact on the most vulnerable elements of the flora is urgently needed for effective management of the Falklands’ National Nature Reserves (NNRs) and 17 recently identified Important Plant Areas (IPAs) [13].

This study is timely because it is only recently that regional climate models (RCMs) have been available [14] and employed to make the first predictions for warming across the Falkland Islands. The Falkland Islands Government is keen to explore useful adaptation and mitigation strategies based on scientific evidence [9]. The last century has seen a 0.5°C increase in annual mean temperature for Stanley, East Falkland [15]. RCMs predict a large increase in the rate of change, with a 1.8 (± 0.34 S.D)°C rise in the mean annual maximum temperature predicted by 2080 [16]. This prediction is based on the A1B emissions scenario, selected because it places a balanced emphasis on all energy sources [17]. The prediction is in line with those made for the A1B emission scenario by the Coupled Model Intercomparison Project multi-model ensemble, which projects increases of 2 to 2.5°C over much of Argentina [17]. In contrast little or no change is predicted for the daily temperature range (DTR; Tmax-Tmin) or annual precipitation [16], although there is currently less agreement across models for changes in precipitation [17] as this variable is more challenging to predict.

Temperature is one of the key climatic variables affecting plant distributions [18, 19, 20, 21]. The mean annual temperature and precipitation of the Falkland Islands is 6.6°C and 559 mm, respectively [16], however, there are significant climatic gradients across the Islands in both temperature and precipitation [16]. Within the Falkland Islands, there is a subset of species with distributions associated with particular temperature ranges [7]; it is these range-restricted species that form the focus of our study.

Species distribution models (SDMs) are widely used to make predictions about the likely impacts of climate change on species’ ranges [22] and as such provide a basis for conservation planning decisions. A modelling approach can also direct further research into fundamental plant biology in understudied areas such as the Falkland Islands by providing testable hypotheses [22]. In the present study we aimed, where possible, to improve the ecological relevance of the component models by incorporating non-climate variables (e.g. water availability, surface geology) that can act as proxies for access to a range of resources at local scales [23, 24, 22]. We also used an ensemble modelling approach (a combination of multiple models) to increase and assess the robustness of our predictions [25, 26].

This is the first investigation into the likely impacts of climate change on plant distributions in the Falkland Islands. We produced species distribution models to predict potential range shifts. We avoided the tendency to apply climate models to all species without regard for whether this application makes logical sense; without knowledge of the physiological tolerances of all potential target species the application of climate models was therefore only appropriate for the subset of species modelled in this study, where there was a clear correlation between distribution and temperature gradients.

## Materials and Methods

### Target Species and Distributions

Our study area comprised the whole of the Falkland Islands archipelago. Given that the climate change predictions for the Falkland Islands are for increased mean annual temperature and no change in mean annual precipitation [16], only species with geographically restricted ranges (identified from Upson and Lewis [7]) that represent archipelago-wide variation in seasonal temperatures (identified from Jones et al. [16]) were appropriate for species distribution modelling to investigate climate change impacts. We omitted species that had fewer than 15 records from the modelling work after finding that these gave poor results; low sample sizes have been shown elsewhere to limit model accuracy [27]. This screening left us with eight target species for the SDM work (Table 1).

**Table 1:** Summary of the species selected for distribution modelling, the number of records available for each (after sampling bias was reduced) and the predictor variables were selected for each.

Species	No. records after reduction in sampling bias	Dates within which records were made	Predictor variables							
			Climatic variable (1 km resolution)				Topographic variables (100 m resolution)			
			Temp. seasonality	Mean temp. of the warmest quarter	Mean temp. of the coldest quarter	Precipitation of the driest quarter	Distance to coast	Summer solar index	Topographic wetness index	Slope
**Upland species**										
*Acaena antarctica* Hook.f.	20	2007–2013	✓	✓						
*Azorella selago* Hook.f.	31	2007–2013	✓	✓					✓	
*Nassauvia falklandica* R. Upson & D.J.N. Hind	20	2009–2013							✓	
**Species with a western distribution**										
*Azorella monantha* Clos	58	2007–2012			✓	✓	✓	✓	✓	
*Blechnum cordatum* (Desv.) Hieron	26	2007–2012			✓	✓				✓
*Sticherus cryptocarpa* (Hook.) Ching	25	2007–2012			✓	✓		✓		
**Species with a southwestern distribution**										
*Nastanthus falklandicus* D.M.Moore	45	2007–2010			✓	✓	✓		✓	
*Plantago moorei* Rahn	30	2007–2009			✓	✓	✓		✓	

We used both observational and vouchered records (obtained from Falkland Conservation and Royal Botanic Gardens (RBG) Kew) that have a resolution of 100 m or finer. Records were gathered between 2007 and 2013—these originate from a range of projects and individuals (details of which can be provided on request) however the majority were collected as part of systematic surveys carried out by R. Upson to identify Important Plant Areas [13].

### Collation of Environmental Variables

The Climate Research Unit at the University of East Anglia (CRU UEA) has recently constructed high spatial resolution climate scenarios for the Falkland Islands and southern Patagonia [16]. Jones et al. (2013) produced reference precipitation and temperature series and used these alongside a set of regional climate models (RCM) developed by CLARIS LPB (http://www.claris-eu.org/) to predict likely climatic changes for the Falkland Islands. The RCMs used were: Rossby Centre RCM R1, 2, 3 (RCA1,2 and 3), Prognastic at Mesoscale (Promes) and MPI-M Regional Model (REMO) [16, 14].

Interpolated climate surfaces for the Falkland Islands were provided by CRU UEA [16] and processed by the RBG Kew Geographic Information Systems (GIS) team (Table 2). These were then standardised (for both geographic projection and cell size) and processed by both UEA and RBG to produce future climate scenarios of these areas for the periods 2011–2040, 2041–2070 and 2071–2100; we refer to these time periods using the years 2020, 2050 and 2080.

**Table 2:** Interpolated climate surfaces produced for the Falkland Islands.

Variable	Comments
BIO1 = Annual Mean Temperature	Included in variable selection process
BIO2 = Mean Diurnal Range (Mean of monthly (max temp—min temp))	Excluded—data artefacts caused by saw tooth of data as seasonality (the months) change as you move across the islands. Giving banding across the islands.
BIO3 = Isothermality (BIO2/BIO7) (* 100)	Excluded—data artefacts caused by saw tooth of data as seasonality (the months) change as you move across the islands. Giving banding across the islands.
BIO4 = Temperature Seasonality (standard deviation *100)	Included in variable selection process
BIO5 = Max Temperature of Warmest Month	Included in variable selection process
BIO6 = Min Temperature of Coldest Month	Included in variable selection process
BIO7 = Temperature Annual Range (BIO5-BIO6)	Included in variable selection process
BIO8 = Mean Temperature of Wettest Quarter	Excluded—Data artefacts caused by saturation at high values causing a plateau of the same high data value
BIO9 = Mean Temperature of Driest Quarter	Excluded—Data artefacts caused by saturation at high values causing a plateau of the same high data value
BIO10 = Mean Temperature of Warmest Quarter	Included in variable selection process
BIO11 = Mean Temperature of Coldest Quarter	Included in variable selection process
BIO12 = Annual Precipitation	Included in variable selection process
BIO13 = Precipitation of Wettest Month	Included in variable selection process
BIO14 = Precipitation of Driest Month	Included in variable selection process
BIO15 = Precipitation Seasonality (Coefficient of Variation)	Excluded—data artefacts caused by saw tooth of data as seasonality (the months) change as you move across the islands. Giving banding across the islands.
BIO16 = Precipitation of Wettest Quarter	Included in variable selection process
BIO17 = Precipitation of Driest Quarter	Included in variable selection process
BIO18 = Precipitation of Warmest Quarter	Included in variable selection process
BIO19 = Precipitation of Coldest Quarter	Included in variable selection process

Interpolated climate surfaces provided by the Climate Research Unit at the University of East Anglia and processed by the Royal Botanic Gardens Kew Geographic Information Systems (GIS) team. All climate variables are at a scale of 1 km.

19 (following those of BIOCLIM [28] see Table 2) climate variables were created based on the UEA dataset [16]. Five climate variables were excluded from the analyses owing to the presence of artefacts (Table 2) which could not be reconciled.

We used ArcGIS 10.0 [29] to generate five non-climate variables from 90 m resolution NASA Shuttle Radar Topographic Mission (SRTM) elevation data processed by CGIAR-CSI [30]. The variables produced were selected to represent resource availability and topographical features that may help define a given species’ ecological niche. Table 3 summarises the non-climate variables considered for inclusion in each distribution model. Elevation itself was not used as it was strongly correlated with the bioclimatic variables.

**Table 3:** Non-climate variables screened for modelling.

Variable	Justification for predictor selection including what ecologically relevant processes they are intended to represent
Surface temperature (solar index)	Surface temperature and access to the resource light. The algorithm used is that of Suggitt et al. [31]; it provides a proxy of clear-day potential solar radiation and is based on that provided by Šúri and Hofierka [32]; this value also accounts for shading.
Water availability (topographic wetness)	Access to water resources. Using the calculations of Bevan and Kirkby [33], where valley bottoms are considered wetter than mountain tops; and flat areas wetter than areas with steep slopes.
Westerly aspect	Level of exposure to prevailing wind (from the west and northwest in the Falkland Islands). Lasseur et al. [34] suggest that the predictive power of aspect performs reasonably well at the local scale (corresponding to 75–175 m^2^ in their study).
Slope angle	An indication of water flow, erosion and soil deposition [34]. Lasseur et al. [34] suggest that the predictive power of slope is optimised at the local scale (75–175 m^2^) [34]. This parameter has also been used as another proxy for general information on water and light access.
Distance to coast	A suite of environmental conditions associated with coastal sites including level of exposure to salt spray and wind.

All non-climate variables are at a scale of 100 m and the top four are based on the Falkland Island digital elevation model.

Sampling bias was reduced by randomly removing all but a single record from each 100 m grid square across the sampled area; this was carried out using the dismo package [35] in R (version 3.0.2) [36]. The sampling for all variables (Table 1) was carried out at 100 m, the resolution of the finest-scale topographic variables.

### Variable Selection

We inspected our dataset both visually (e.g. through plotting data as boxplots, histograms and regression lines) as well as via relevant statistical analyses. If two variables were highly correlated (r > 0.7 or < -0.7, Pearson product-moment correlations) we kept the predictor which is highlighted in the literature and/ or known through expert opinion to be ecologically important to a species. We then used the automatic step-wise approach (applied to a generalized linear model with a binomial distribution) using the R function step() and assessed the model quality based on the Akaike information criterion (AIC) value. Following the 10:1 (data points: number of variables) rule of thumb [37,38,39] to avoid over-fitting, we selected those variables with the lowest AIC scores. Table 1 details the variables selected for modelling the distribution of each target species.

### Model and Ensemble Development

We used five modelling algorithms to determine the current environmental ranges of each species, using a resolution of 100 m and a geographical extent covering the entire Falkland Islands. General linear models (GLM), general additive models (GAM), generalised boosting models (GBM), Random Forest (RF) and Maximum Entropy Distribution Modelling (MaxEnt), were all produced using the R statistical package BIOMOD2 [40, 25, 41]. The species data comprised presence records plus randomly generated data points (referred to as pseudo-absences), so that the latter were 10 times the former in number. The pseudo-absences cover all available environmental space across the Falkland Islands and therefore a small proportion may in theory include environmental conditions that are also suitable for the target species [42,43,44]. An 80:20 split was randomly performed ten times to partition these data into training and testing data, respectively [45]. These data sub-samples were used to assess the relative importance of variables used in each developed model, model sensitivity and specificity, as well as overall model performance [45].

Assessment of variable importance is vital in fully understanding each species distribution model as it gives a useful indication of which variables are driving the model. Variable importance was established using the ‘random shuffle’ method implemented in the BIOMOD2 package [40]; the importance of a variables is assessed by determining how well the model performs when in turn, data associated with each variable, are randomly shuffled.

A range of techniques were employed within BIOMOD2 [40] to assess the predictive performance of each model [46, 47], however assessments of all models primarily considered the True Skill Statistic (TSS) [48, 49] measure. This is because it retains the advantages of Kappa by accounting for both sensitivity and specificity and has been shown to be independent of prevalence, an inherent problem with Kappa statistics [49]. Final models, incorporating all the available data points [50], were used to generate maps of the overall modelled areas. By convention, an algorithm is considered excellent if the average TSS/ Kappa score across all model replicates is >0.8 and good if 0.6–0.8. For each species only those models with an average TSS score above 0.7 were included in ensemble-model building. The ensemble process undertaken within the BIOMOD2 framework, produced ensemble forecast maps for each species that were based on weighted means and covered the whole of the Falkland Islands. We used TSS scores for final target species ensemble model assessment (Table 4).

**Table 4:** Confusion matrix tables for TSS-weighted mean ensemble models for each target species.

Dsitribution	Species	Prediction	Observed number (proportion)		Model Accuracy (TSS)	Total area predicted to be suitable (km^2^)
			Present	Pseudo-absent		
Upland	*Acaena Antarctica* Threshold = 400	Present	20	4	97.8	201
		Pseudo-absent	0	176		
	*Azorella selago* Threshold = *476*	Present	31	0	100	175
		Pseudo-absent	0	270		
	*Nassauvia falklandica* Threshold = *648*	Present	20	0	100	154
		Pseudo-absent	0	176		
Western	*Azorella monantha* Threshold = *266*	Present	58	14	97.3	481
		Pseudo-absent	0	514		
	*Blechnum cordatum* Threshold = *283*	Present	26	5	97.8	757
		Pseudo-absent	0	225		
	*Sticherus cryptocarpa* Threshold = *367*	Present	25	6	97.2	555
		Pseudo-absent	0	211		
Southwestern	*Nastanthus falklandicus* Threshold = *292*	Present	45	12	97.2	396
		Pseudo-absent	0	412		
	*Plantago moorei* Threshold = 415	Present	30	4	98.1	302
		Pseudo-absent	0	265		

These data summarise the true positives, false positives, true pseudo-absences and false pseudo-absences. Cut-off threshold values indicated. Also displayed is one measure of accuracy for each ensemble model: True Skill Statistic (TSS) and also the total area predicted to be suitable.

### Modelling Future Changes to Species Distributions

Using BIOMOD2 [26] and the mapped climatic predictions for 2020, 2050 and 2080 we projected our ensemble models for present day (2013) potential distributions of species to these time periods.

Distribution maps were converted to presence-absence maps using a species-specific thresholds that maximised the sensitivity-specificity sum [51], one of the most effective and objective methods for minimising both the false presence and false absence rates [52, 53].

We used these presence-absence maps to derive the predicted present day and future environmentally suitable area. Three range size variables were calculated: % change in environmentally suitable area, % overlap in environmentally suitable area and % change in mean environmental suitability (described below and following the methodology of Cabrelli et al. [54]). Significance testing was carried out where there were different climate values from multiple RCMs (and therefore not possible using present day data). The aim was to determine whether variation between RCMs influenced model predictions. The Wilcoxon rank-sum test was used to test for significant differences between 2020 and 2080 data.

### Change in Predicted Species Distributions

We calculated the change in environmentally suitable area between the current climate presence-absence map and those for 2020, 2050 and 2080. This was calculated as a reduction or increase in relation to the present day number of thresholded species ‘present’ grid cells. This was repeated for each RCM and a mean was calculated for each species and each time frame [54].

We also calculated the percentage overlap between current and future predicted environmentally suitable area using the presence-absence maps and again calculated the mean value across the RCMs [54], as this predicts resilience to the specified change.

To calculate the percentage change in overall environmental suitability we followed the method outlined by [54]; using the non-thresholded current and future maps, we calculated the mean environmental suitability for our study area, the entire Falkland Islands, for each time period. These values were used as a measure of environmental suitability for each time period. The change from the present to each time period was expressed as a percentage so that a percentage change of less than 100% is indicative of a decrease in environmental suitability over time (indicating a high vulnerability for the species to climate change) whereas a change greater than 100% indicates increased environmental suitability (low vulnerability of the species to climate change).

Likely persistence of present day target species populations was tracked through the 2020, 2050 and 2080 mean environmental suitability maps (generated as described above). This point analysis [55] enabled calculation of the proportion of sites predicted to remain suitable for each time period.

### Overlap of Environmentally Suitable Area with Protected Areas Network under Current and Predicted Climate Change

The relative effectiveness of the current legally protected areas, National Nature Reserves (NNR), for plant conservation, in the face of climate change, was assessed against that of identified Important Plant Areas (IPAs) [13, 56]. We compared the presence-absence maps with GIS layers of the NNRs and IPAs and calculated the percentage change in the number of environmentally suitable grid cells that fell within these conservation areas for each of the future maps for each species. This analysis was carried out solely on those species predicted to be at risk of extinction, i.e. we did not do this for western target species.

We identified species that showed a decrease, by 2080, in the number of environmentally suitable grid cells overlapping with conservation areas. For these species presence-absence maps were used to identify potential refugia sites, i.e. sites that are predicted to remain or become environmentally suitable for each species by 2080.

## Results

### Model Assessment

**Fig 1:**
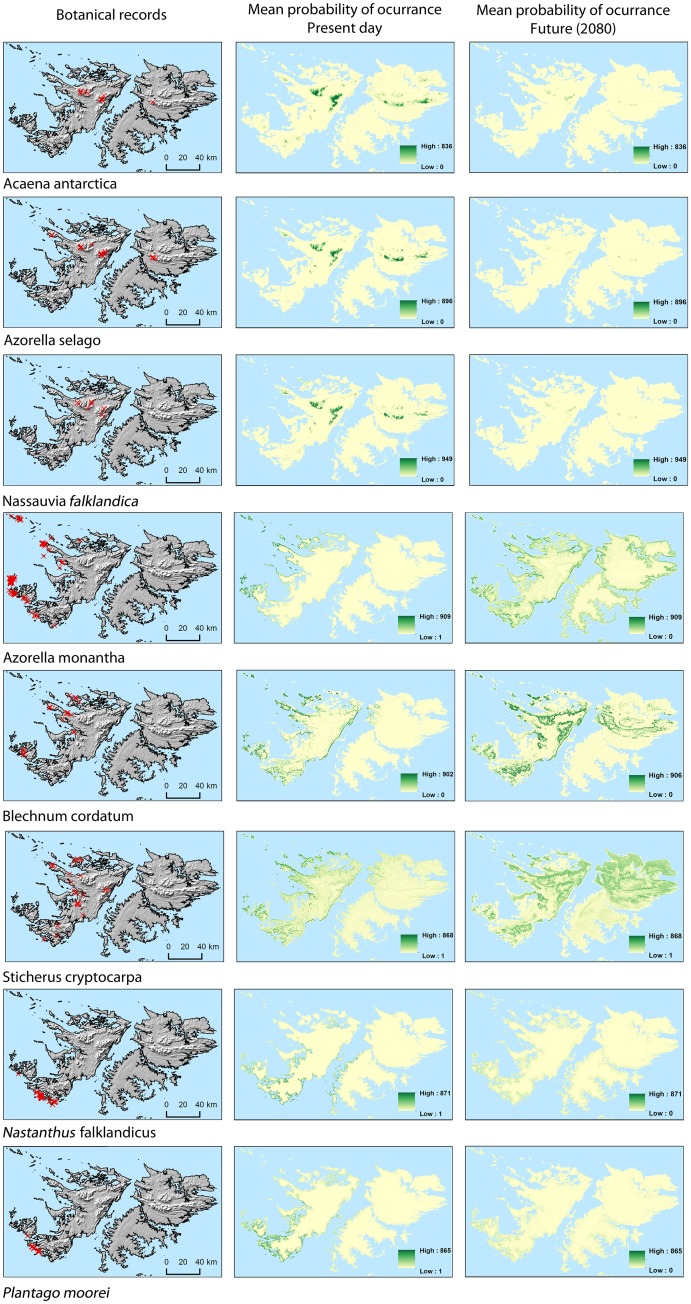
Target species’ records and mean probability of occurrence under current and future (2080) climate scenarios. Means are calculated across five Regional Climate models. Mean probability of occurrence is based on ensemble models for upland species *Acaena antarctica*, *Azorella selago*, *Nassauvia falklandica*, western species *Azorella monantha*, *Blechnum cordatum*, *Sticherus cryptocarpa* and southwestern species *Nastanthus falklandicus*, *Plantago moorei*.

**Fig 2:**
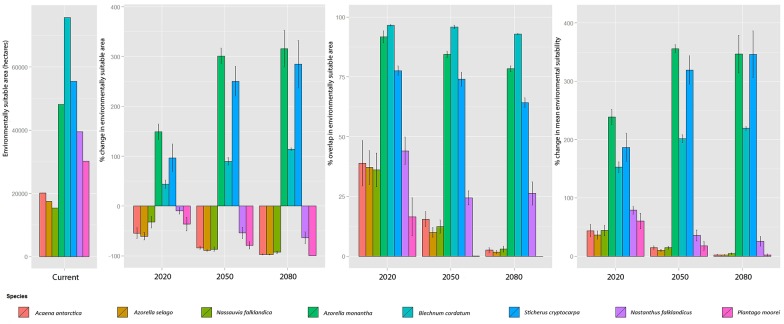
Target species’ present day environmentally suitable space and predicted changes in short, medium, long term. In relation to the present day, predictions are shown for the mean (± 1 S.E) percentage by which the environmentally suitable area available changes (negative values correspond to a range reduction, positive to an expansion), percentage overlap in environmentally suitable area and percentage change in mean environmental suitability; changes are calculated for the short (2020), medium (2050) and long term (2080). Mean values calculated for each species across the five RCMs used. It is worth noting that % overlap in suitable environmental space would be 100% for the present day. For changes in environmentally suitable area available a value of < 100%, 100% or > 100% corresponds to a decrease, no alteration or increase.

Amongst target species and under today’s climate, upland species have the smallest predicted ranges, whereas western species have the largest predicted ranges (Table 4, Figs 1 and 2). Overall, ensemble model accuracy remained very high, ranging from 97.2–100% (Table 4). Table 5 shows the variables most important in the modelling of each species’ distribution for each model type: for all upland species’ the most important contributing variable in the models is mean temperature of the warmest quarter.

**Table 5:** A summary of the relative importance of the most important variables for each species and model.

Distribution	Species	Model variable	Model variable influence (%)				
			GLM	GAM	Maxent	RF	GBM
Upland	***Acaena antarctica***	Mean T°C Warmest Quarter	100.0	93.1	69.4	66.3	84.9
	***Azorella selago***	Mean T°C Warmest Quarter	91.5	99.7	48.9	75.5	97.3
	***Nassauvia falklandica***	Mean T°C Warmest Quarter	70.6	89.5	70.1	64.7	89.0
Western	***Azorella monantha***	Mean T°C Coldest Quarter	47.6	52.0	31.1	55.9	46.2
	***Blechnum cordatum***	Slope angle	54.8	69.7	51.1	52.2	61.0
		Mean T°C Coldest Quarter	38.6	29.6	26.7	41.8	37.3
	***Sticherus cryptocarpa***	Surface temperature	79.9	73.3	74.0	65.2	84.4
		Mean T°C Coldest Quarter	26.1	30.8	34.8	33.9	22.7
Southwestern	***Nastanthus falklandicus***	Distance to Coast	48.7	54.1	33.0	33.6	31.6
		Mean T°C Coldest Quarter	29.3	20.5	29.1	25.1	29.1
	***Plantago moorei***	Mean T°C Coldest Quarter	39.1	34.2	*	39.4	43.7
		Distance to Coast	*	*	32.6	*	*

For the most important variables, this is a summary of their relative importance for a given model. Results are presented per species per model. The importance of each variable is 100 minus the percentage correlation score between the original prediction and the prediction made with a randomly shuffled variable. So the higher the value the greater the importance of a given variable to that model.

*Not most important variable.

For the western species the most important contributing variable in the models varies, however mean temperature of the coldest quarter has either the most or high influence for all. For the ferns *Blechnum cordatum* and *Sticherus cryptocarpa*, slope and solar radiation are ranked as most important (Table 5).

For the southwestern species *Nastanthus falklandicus*, distance to coast, then mean temperature of the coldest quarter have the most influence on all component models, followed by precipitation of the driest quarter and water availability. For *Plantago moorei* four of the five component models rank mean temperature of the coldest quarter as the most important variable and three out of four agree on distance to coast as of least influence. The best Maxent model for *P*. *moorei* also placed very similar importance (32.5%) on mean temperature of the coldest quarter compared to the other models, however it narrowly ranked distance to coast as the most important variable (32.6%).

Fig 1 shows the current environmentally suitable areas for each target species using the ensemble modelling approach. Overall the model predictions match well with current known distributions. The largest discrepancy between the current known distribution and model predictions occurs for the western species *B*. *cordatum* and *S*. *cryptocarpa*; high probabilities of occurrence for these species are predicted on the east coast of West Falkland (Fig 1), where neither species is currently known to occur. There is also a difference in both southwestern species which have ranges predicted well beyond their current known distributions.

### Future Range Change Predictions

There is agreement across ensemble SDMs for each RCM that there will be a decline in the environmental range for all upland and southwestern species across all time periods (Fig 2). For the upland species *Acaena antarctica*, *Azorella selago* and *Nassauvia falklandica*, range contractions of 98%, 97% and 93%, respectively, are predicted by 2080 in relation to their current potential ranges. In addition Wilcoxon rank-sum significance tests (using the five RCM results as replicates) between 2020 and 2080 show decreases of 95% [*P* = 0.045], 93% [*P* = 0.008] and 89% [*P* = 0.008] for the same species (Fig 2); this indicates that the current ranges for these species will be almost completely lost by 2080. The decline in upland species’ predicted environmental ranges is caused by range contraction rather than range shift (Fig 1) leading to overlap figures of only 3%, 2% and 3% for *Acaena antarctica*, *Azorella selago* and *Nassauvia falklandic* by 2080, respectively (Fig 2). The majority of areas that satisfy the environmental niches required for these upland species are predicted to be lost by 2080, with declines in the mean percentage suitability across the Islands between 2020 and 2080 of 96% [*P* = 0.036], 94% [*P* = 0.008] and 88% [*P* = 0.008] for *Acaena antarctica*, *Azorella selago* and *Nassauvia falklandica*, respectively (Fig 2).

Between 2020 and 2080 the western species *Azorella monantha*, *B*. *cordatum* and *S*. *cryptocarpa* are predicted to increase their range size by 67% [*P* = 0.016], 49% [*P* = 0.008] and 96% [*P* = 0.016], respectively (Fig 2). Alongside this the majority of the currently suitable sites are predicted to remain so for all three species until 2080. The overall mean environmental suitability is predicted to increase (Fig 2), indicating that these species could benefit from climate change. From 2020 to 2080 mean environmental suitability is predicted to increase by 45% [*P* = 0.05556], 44% [*P* = 0.008], 85% [*P* = 0.016] for *Azorella monantha*, *B*. *cordatum* and *S*. *cryptocarpa*, respectively. *Azorella monantha* is a coastal species (Fig 1) which is predicted to increase its range eastwards with the most suitable sites predicted to remain close to the coast (Fig 1). *B*. *cordatum* is predicted to expand inland and altitudinally as well as into northern parts of East Falkland (Fig 1). *S*. *cryptocarpa* is predicted to follow a similar trend as *B*. *cordatum* although with a greater area overall predicted to be suitable (Figs 2 and 1).

Of the southwestern species, the more range-restricted *P*. *moorei* is predicted to be more vulnerable to the predicted temperature increase than *Nastanthus falklandicus* (Fig 2). 26% of the present day suitable areas are predicted to remain suitable for *Nastanthus falklandicus* in the long term (Fig 2); there is a significant decrease in the area predicted to remain suitable for *Nastanthus falklandicus* between 2020 and 2080 [*P* = 0.016] (Fig 2). In contrast the present-day environmentally suitable areas for *P*. *moorei* are not predicted to remain suitable in the long term, with the mean percentage overlap between the present day and 2080 predicted to be zero. In addition, the overall mean environmental suitability for *P*. *moorei* is predicted to decrease by 96% from 2020 to 2080 [*P* = 0.012] (Fig 2).

### Likely Persistence of Current Populations under Predicted Climate Change

The upland species show a substantial decline in likely persistence of current populations over time. There is also a general decline, but at varying rates, for the two southwestern species, whilst in contrast the western species remain highly persistent (Table 6).

**Table 6:** The percentage of currently known populations of target species that overlap/ are predicted to overlap with environmentally suitable space across the Falkland Islands in the face of predicted temperature increases.

Distribution		Percentage overlap			
		Present day	2020	2050	2080
**Upland**	***Acaena antarctica***	100	47	10	0
	***Azorella selago***	100	68	51	0
	***Nassauvia falklandica***	100	17	0	0
**Southwestern**	***Nastanthus falklandicus***	100	79	51	46
	***Plantago moorei***	100	12	0	0
**Western**	***Azorella monantha***	85	89	86	86
	***Blechnum cordatum***	84	78	80	78
	***Sticherus cryptocarpa***	78	56	49	39

No known locations of upland species or southwestern species are predicted to remain environmentally suitable in the face of predicted climate change (Table 6). Several locations across a restricted part of the south-east coast of East Falkland are predicted to be suitable for *P*. *moorei*, however no populations currently occur in this area. For *Nastanthus falklandicus* 46% of currently known sites are predicted to remain environmentally suitable in the long term (Table 6).

In the medium term no current sites are predicted to remain environmentally suitable for *Nassauvia falklandica or Plantago moorei*, but 10% and 51% of the currently known sites for *Acaena antarctica* and *Azorella selago*, respectively, are predicted to remain environmentally suitable (Table 6). In the short term, however, a varying proportion of current sites are predicted to remain suitable for each species.

### Overlap of Environmentally Suitable Space with Protected Areas Network under Current and Predicted Climate Change

At present there are no environmentally suitable areas for upland species that occur within National Nature Reserves in the Falkland Islands and this does not change under the predicted temperature increases (S1 Appendix). In contrast identified Important Plant Areas encompass 67%, 43% and 48% of the present-day predicted suitable environmental space for *Acaena antarctica*, *Azorella selago* and *Nassauvia falklandica*, respectively (S1 Appendix). The areas predicted to remain environmentally suitable that fall within IPAs are predicted to decrease significantly from 2020 to 2080 for *Acaena antarctica* [*P* = 0.016], *Azorella selago* [*P* = 0.009] and *Nassauvia falklandica* [*P* = 0.009].

Though there are no currently occupied sites that will remain suitable in the future for upland species, there are potential refugia sites within all three of the major upland ranges of the Falkland Islands: the Hornby Mountains (West Falkland), Hill Cove Mountains (West Falkland) and Wickham Heights (East Falkland). By 2080 suitable areas are predicted for *Azorella selago* and *Nassauvia falklandica* within all three major hill ranges, whilst *Acaena antarctica* is only predicted to occur within the Hornby Mountains. Notably for *Nassauvia falklandica*, a species not currently known from East Falkland, modelling predicts suitable environmental conditions in 2080 along two sections of the major hill range on this island, Wickham Heights.

The IPA network currently covers 59% and 49% of the present-day predicted suitable area for the southwestern species *P*. *moorei* and *Nastanthus falklandicus*, respectively, while NNRs cover 14% and 18%, respectively (see supplementary material). The environmentally suitable area that falls within IPAs is not predicted to decrease significantly between 2020 and 2080 for *Nastanthus falklandicus* [*P* = 0.251] but is predicted to significantly decline for *P*. *moorei* [*P* = 0.005]. A significant decline in the environmentally suitable area covered by NNRs is predicted for both *Nastanthus falklandicus* [*P* = 0.009] and *P*. *moorei* [*P* = 0.005] from 2020 to 2080, with this predicted to decline to zero by 2080 for *P*. *moorei*.

## Discussion

Our results demonstrate the vulnerability of a remote oceanic island to climate change impacts, showing for the first time that significant range shifts are predicted amongst the native flora of the Falkland Islands under the predicted warming. Our results highlight in particular the vulnerability of a suite of species from upland and coastal areas of the Falkland Islands, implying the need for management strategies that do not place additional stresses on these landscapes. Similar to other South Atlantic islands [57,58,59,60], the Falklands hold a relatively small flora with likely lower levels of diversity amongst plant functional groups and therefore lower levels of redundancy within its ecosystems. It may as a result be inherently more vulnerable to environmental change than mainland areas and islands with higher diversity. Retaining maximum plant diversity is therefore vital to ensuring maintenance of plant functional diversity with this in turn promoting increased resilience to the effects of climate change [61] which is key to the survival of this unique flora.

### Potential Impacts on Upland Species

All model outputs agree that the present day ‘upland environmental niche’ is predicted to decrease and that this is likely to have a significantly negative impact on species restricted to these areas across the Falklands. This is directly related to predicted temperature increases and therefore migration to higher altitudes may be the only option for survival [62]. Potential upland refugia sites are identifiable however it is important to note that those predicted to remain suitable in the long term do not currently hold populations of target species. For example, *Nassuavia falklandica* is known only from West Falkland but the highest point on the Islands, on East Falkland, is predicted to be one of the locations best able to maintain suitable environmental conditions for this species. With limited available sites, consideration should therefore be given early on to translocation experiments and the role of assisted migration [63, 64, 65, 66]. To allow this and other potential management options to be investigated, seed collecting and banking efforts should be expanded alongside the production of horticultural protocols to better understand upland species’ growth requirements and preferences.

Considering sites where these particularly sensitive species currently grow, “every effort should be made to minimize other stressors on population viability and to monitor population trends” [67]. A re-assessment of current management practices across these and potential long term upland refugia sites would enable an assessment of where threats are likely to be causing unnecessary additional stress to vulnerable communities. In the context of the majority of land in the Falkland Islands being privately owned and used for livestock grazing, a first stage could be the identification and prioritisation of assessments of upland sites that are government owned, currently ungrazed or of low grazing potential but of high value in terms of their potential to act as refugia for vulnerable upland species. Such management considerations are particularly pressing on isolated Islands such as the Falkland Islands where appropriate sites are limited.

A greater understanding of the ecology of vulnerable species is necessary to better inform management decisions and continue to refine model predictions. Considering the target upland species investigated here, there have been no previous studies into the ecology of *Acaena antarctica* or the recently described *Nassauvia falklandica* [68]. Research on *Azorella selago* at its sub-Antarctic locations indicates a sensitivity to drought and competition from faster growing species [69] therefore making it at risk from both direct and indirect impacts of climate change. In the Falklands, *A*. *selago* is largely confined to upland sloped sites with reasonable drainage and those experiencing lower temperatures—mean temperature of the warmest quarter and water availability drive its distribution in the models presented here. Similar to the current study, this cushion plant is predicted to move up altitudinal gradients in response to warming on sub-Antarctic Marion Island, where it is a keystone species [70].

### Potential Impacts on Southwestern and Western Species

Modelling of two southwestern endemic species *Nastanthus falklandicus* and *P*. *moorei* indicate a more extreme response to the predicted temperature increase by the latter, owing perhaps to their limited distribution. The incorporation of soil data may improve predictions for these species in particular as they are largely restricted to sandy, well-drained soils and open habitats with a saline influence [13]. Predictions for *Nastanthus falklandicus* indicate that almost half of the sites where it currently grows are likely to remain environmentally suitable in the long term—this is encouraging but does not take into account current additional stresses placed on these sites such as high levels of erosion that may be exacerbated from livestock impacts. This again highlights the need to review the management of key biodiversity sites across the Islands and work towards implementing the most sustainable practices to maximise the chances of plants being able to track climate change.

All target western species, *Azorella monantha*, *S*. *cryptocarpa* and *B*. *cordatum*, are predicted to experience an expansion in suitable environmental space to varying degrees, owing to their current preference for areas with a milder climate, including higher mean annual temperatures and higher temperatures in the coldest quarter. Vulnerability to frost during the main growing season should be investigated as this may play an important part in explaining their current westerly distributions and restriction to lower altitudes, but it is clear that research into physiological tolerances of the flora of the Falkland Islands is urgently needed to better understand the possible short and medium term responses to climate change. In general there is a greater need for fundamental research into the basic biology, such as germination requirements, of the species that form this unique flora.

### The Role of NNRs and IPAs

We looked at the predicted future overlap between conservation areas and target species’ ranges for both NNRs and IPAs because the former are legally protected, but the majority were not selected with plant conservation in mind. In contrast, Important Plant Areas have been specifically identified to capture the most important areas for plants across the Islands [13, 56]. Our results clearly show the usefulness of recognising IPAs as vital to long term conservation of threatened plant species in the face of climate change but also highlight areas that should be considered for addition to the network as potential refugia from a changing climate. The effectiveness of IPA networks ultimately depends on how sites are used with respect to current and future management options. The Protected Area network in the Falkland Islands is currently under review and the potential impacts of climate change are high on the list of factors being considered in evaluating the long-term conservation of biodiversity in the Falkland Islands [9].

### Model Evaluation

One major challenge when modelling range-restricted species is the lower number of records available [71]. Confidence in the models relates to confidence in our knowledge of the distribution of these species and in capturing the major drivers of broad-scale trends in their distributions.

We found that ensemble models for all eight species provided useful predictions of distribution as defined by TSS values greater than 0.7. All target species’ models had consistently strong performances, high evaluations and similar results between the component models. High model performance for species with lower frequencies as found in this study can in part be interpreted as reflecting the fact that they are niche specialists rather than generalists [72].

Despite this, areas predicted to be suitable under the current climate include sites where species do not presently occur. This is particularly apparent for *B*. *cordatum* and the southwestern species. This could be for a number of reasons; the sites may be unsuitable due to other variables that could not have been included in the current models (such as soil type); or perhaps they once were present and now are not as a result of deleterious management practices [73,74]. As further information on edaphic factors, such as soil type, become available these should be included into models and tests carried out to assess any reduction in false positives. In addition predicted range changes are based on modelled changes in environmentally suitable space and therefore actual range changes will be impacted by additional factors including dispersal ability and competitive advantage at new proposed suitable sites.

We are aware that our modelling is limited by data availability and that additional variables, such as mapped soil types, may have been useful for better characterising each species’ ecological niche. Additionally confounding effects may come from the differing management histories across the Islands, in particular in relation to grazing levels. An increase in the number of weather stations and better representation of inland sites would also improve the climate surface maps [14]. Running RCMs with other emissions scenarios would also improve our understanding of the likely range in terms of climatic changes.

### Application of Distribution Modelling Results

Our research allows appropriate selection of sites across local temperature gradients for the establishment of both observational and experimental studies. An integrated approach at the outset would help to address the limitations associated with each method, with observational data allowing us to verify experimental results (e.g. [75]) and experiments helping to improve our mechanistic understanding of responses to climate change [76, 77]. Future sites should focus on altitudinal and East-West temperature gradients across the archipelago and address questions regarding both target species’ and community responses to climate change. Plant species respond in different ways to environmental change and hence climate change can alter community composition [78, 79, 80, 81]. Long term observational data gathered from altitudinal transects can, for example, monitor whether upland cushion heath habitats (that include target upland species) are vulnerable to encroachment by grassland as observed on Marion Island [82] and therefore potentially inform management decisions of key sites. Appropriately selected sites covering the temperature gradient from East to West Falkland could investigate predictions made with regard to species range expansions or contractions.

Experimental warming plots have been set up at one site on East Falkland within dwarf shrub heath (a dominant habitat type across the Islands) and a coastal *Festuca*-*Poa* community (fairly restricted) [83]. The mean increase in soil temperature facilitated in the experimental warming carried out by Bokhorst et al. [83], is close to the average warming predicted for the short term (*c*. 0.2°C per decade on average), suggesting this open top chamber design could be further employed. Bokhorst et al. [83] suggest that small increases in temperature in the Falklands may quickly lead to decreased soil moisture leading to ‘more stressful conditions for plants’–in the case of the open grassland community this led (in addition to a dry summer the year before) to a decrease in total vegetation cover and the loss of two species. An expansion of experimental sites to habitats that contain particularly vulnerable species (such as upland cushion heath) is urgently needed.

### Concluding Remarks

We have shown that with careful selection of target species, distribution modelling can provide invaluable insights into the likely impacts of climate change on species’ ranges across isolated oceanic islands. Moreover our results provide the means to select appropriate places for vegetation monitoring. Combining an expansion of artificial warming experiments with such observational studies across natural gradients would deepen our understanding of the likely impacts of climate change. This approach and the emerging results from this study are of wider application to other UK Overseas Territories as well as other Small Island Developing States who are facing similar challenges in conserving their biodiversity and meeting their agreed commitments to multi-lateral environments agreements.

## Supporting Information

S1 Table Overlap between environmentally suitable sites for species at risk of extinction and National Nature Reserves or Important Plant Areas. Percentage overlap between environmentally suitable sites for species at risk of extinction and National Nature Reserves (NNRs) or Important Plant Areas (IPAs) under the current climatic conditions and those predicted in the short, medium and long-term.
(DOCX)
