# Supplementary material for: Potential Impacts of Climate Change on Native Plant Distributions in the Falkland Islands
Source: PLoS One. 2016 Nov 23;11(11):e0167026. doi: 10.1371/journal.pone.0167026 (PMC5120834; doi:10.1371/journal.pone.0167026)
Supplement: S1 Table — Percentage overlap between environmentally suitable sites for species at risk of extinction and National Nature Reserves (NNRs) or Important Plant Areas (IPAs) under the current climatic conditions and those predicted in the short, medium and long-term. (DOCX) [file pone.0167026.s001.docx]

# Supporting Information

## S1 Appendix

Results indicating importance of recognising Important Plant Areas in addition to the current protected areas network of the Falkland Islands; the table shows percentage overlap between environmentally suitable sites for species at risk of extinction and National Nature Reserves (NNRs) or Important Plant Areas (IPAs) under the current climatic conditions and those predicted in the short, medium and long-term.

|  | **Time period** | ***Acaena antarctica*** | ***Azorella selago*** | ***Nassauvia falklandica*** | ***Nastanthus falklandicus*** | ***Plantago moorei*** |
| --- | --- | --- | --- | --- | --- | --- |
| **Percentage of environmentally suitable geographical space that overlaps with NNR network (km^2^)** | **Present day** | 0 | 0 | 0 | 18.47 | 14.00 |
|  | **2020** | 0.00 (±0.00) | 0.00 (±0.00) | 0.00 (±0.00) | 20.18 (±0.53) | 8.48 (±0.66) |
|  | **2050** | 0.00 (±0.00) | 0.00 (±0.00) | 0.00 (±0.00) | 10.25 (±0.86) | 3.58 (±0.52) |
|  | **2080** | 0.00 (±0.00) | 0.00 (±0.00) | 0.00 (±0.00) | 7.06 (±0.85) | 0.00 (±0.00) |
| **Percentage of environmentally suitable geographical space that overlaps with identified IPA network (km^2^)** | **Present day** | 67.71 | 43.49 | 48.11 | 49.02 | 58.55 |
|  | **2020** | 19.86 (±1.93) | 17.17 (±1.06) | 20.86 (±1.40) | 34.59 (±0.75) | 9.54 (±1.24) |
|  | **2050** | 8.15 (±0.94) | 5.72 (±0.41) | 7.27 (±0.67) | 24.62 (±1.25) | 0.40 (±0.06) |
|  | **2080** | 2.59 (±0.48) | 0.97 (±0.11) | 1.95 (±0.32) | 29.10 (±2.22) | 0.00 (±0.00) |

# Supporting Information

## S1 Appendix

Results indicating importance of recognising Important Plant Areas in addition to the current protected areas network of the Falkland Islands; the table shows percentage overlap between environmentally suitable sites for species at risk of extinction and National Nature Reserves (NNRs) or Important Plant Areas (IPAs) under the current climatic conditions and those predicted in the short, medium and long-term.

|  | **Time period** | ***Acaena antarctica*** | ***Azorella selago*** | ***Nassauvia falklandica*** | ***Nastanthus falklandicus*** | ***Plantago moorei*** |
| --- | --- | --- | --- | --- | --- | --- |
| **Percentage of environmentally suitable geographical space that overlaps with NNR network (km^2^)** | **Present day** | 0 | 0 | 0 | 18.47 | 14.00 |
|  | **2020** | 0.00 (±0.00) | 0.00 (±0.00) | 0.00 (±0.00) | 20.18 (±0.53) | 8.48 (±0.66) |
|  | **2050** | 0.00 (±0.00) | 0.00 (±0.00) | 0.00 (±0.00) | 10.25 (±0.86) | 3.58 (±0.52) |
|  | **2080** | 0.00 (±0.00) | 0.00 (±0.00) | 0.00 (±0.00) | 7.06 (±0.85) | 0.00 (±0.00) |
| **Percentage of environmentally suitable geographical space that overlaps with identified IPA network (km^2^)** | **Present day** | 67.71 | 43.49 | 48.11 | 49.02 | 58.55 |
|  | **2020** | 19.86 (±1.93) | 17.17 (±1.06) | 20.86 (±1.40) | 34.59 (±0.75) | 9.54 (±1.24) |
|  | **2050** | 8.15 (±0.94) | 5.72 (±0.41) | 7.27 (±0.67) | 24.62 (±1.25) | 0.40 (±0.06) |
|  | **2080** | 2.59 (±0.48) | 0.97 (±0.11) | 1.95 (±0.32) | 29.10 (±2.22) | 0.00 (±0.00) |
